# Cytotoxic pathways activated by multifunctional thiosemicarbazones targeting sigma-2 receptors in breast and lung carcinoma cells

**DOI:** 10.1007/s43440-023-00531-y

**Published:** 2023-10-05

**Authors:** Joanna Kopecka, Alessandra Barbanente, Daniele Vitone, Fabio Arnesano, Nicola Margiotta, Paola Berchialla, Mauro Niso, Chiara Riganti, Carmen Abate

**Affiliations:** 1 Department of Oncology, University of Turin, via Nizza 44, 10126 Turin, Italy; 2 Dipartimento di Chimica, Università degli Studi di Bari Aldo Moro, Via Orabona 4, 70125 Bari, Italy; 3 Department of Clinical and Biological Sciences, University of Turin, via Santena5/bis, 10126 Turin, Italy; 4 Dipartimento di Farmacia-Scienze del Farmaco, Università degli Studi di Bari Aldo Moro, Via Orabona 4, 70125 Bari, Italy; 5 Consiglio Nazionale delle Ricerche (CNR), Istituto di Cristallografia, Via Amendola, 70125 Bari, Italy

**Keywords:** Thiosemicarbazones, Adenocarcinoma breast tumor, Adenocarcinoma lung tumor, Sigma receptor, Caspase 3/7/9, ER and mitochondrial stress

## Abstract

**Background:**

Multifunctional thiosemicarbazones (TSCs) able to bind sigma receptors and chelate metals are considered as a promising avenue for the treatment of pancreatic cancer due to the encouraging results obtained on in vitro and in vivo models. Here, we assessed the biochemical mechanism of these TSCs also on lung (A549) and breast (MCF7) cancer cells.

**Methods:**

The density of sigma-2 receptors in normal (BEAS-2B and MCF10A) and in lung and breast (A549 and MCF7) cancer cells was evaluated by flow cytometry. In these cells, cytotoxicity (MTT assay) and activation of ER- and mitochondria-dependent cell death pathways (by spectrofluorimetric assays to measure Caspases 3/7/9; qRT-PCR detection of GRP78, ATF6, IRE1, PERK; MitoSOX, DCFDA-AM and JC-1 staining), induced by the TSCs FA4, MLP44, PS3 and ACThio1, were evaluated.

**Results:**

FA4 and PS3 exerted more potent cytotoxicity than MLP44 and ACThio1 in all cancer cell lines, where the density of sigma-2 receptors was higher than in normal cells. Remarkably, FA4 promoted ER- and mitochondria-dependent cell death pathways in both cell models, whereas the other TSCs had variable, cell-dependent effects on the activation of the two proapoptotic pathways.

**Conclusions:**

Our data suggest that FA4 is a promising compound that deserves to be further studied for lung and breast cancer treatment. However, the other multifunctional TSCs also hold promise for the development of therapies towards a personalized medicine approach. Indeed, the presence of the sigma-2 receptor-targeting moiety would lead to a more specific tumor delivery embracing the characteristics of individual tumor types.

**Supplementary Information:**

The online version contains supplementary material available at 10.1007/s43440-023-00531-y.

## Introduction

There is a pressing need for alternative therapeutic strategies to face tumors, considered the secondary cause of death in the United States after cardiovascular diseases. According to the 2023 Cancer Statistics Report [1], lung cancer is the leading cause of cancer death in both men and women aged 50 and older, far more lethal than breast cancer. However, the leading cause of cancer death in women aged 49 is still breast cancer, which has the highest incidence rate in women. Despite the still elusive mechanism of action, the sigma-2 receptor, which is overexpressed in several tumors, is emerging as a target for cancer treatment, with breast and lung cancer cells characterized as responsive to sigma-2 receptor-mediated cytotoxic action [2–7]. Thus, an already known class of thiosemicarbazones (TSCs) binding sigma-2 receptors, which demonstrated a very encouraging antitumor profile in vitro and in vivo in several pancreatic cancer models [3, 8, 9], was here tested in breast MCF7 and lung A549 cells. These TSCs were produced according to a MultiTarget Directed Ligands (MTDL) [10] approach with the aim of simultaneously chelating metals and modulating the drug efflux pump P-glycoprotein (P-gp) together with sigma-2 receptors [3, 8] to obtain a synergistic effect. This strategy, which led to potent cytotoxic TSCs, was based on the efficacy of some sigma-2 receptor ligands against highly aggressive tumors [8, 11–14] and on the sensitivity of cancer cells to changes in energy levels and needs [2]. In fact, the interaction with the subtype 2 of the sigma receptors activates apoptotic pathways closely related to the type of cell and molecule, while, upon chelation of the metal (iron or copper), TSCs are able to alter the cellular energy balance preventing cancer cell proliferation.

**Fig. 1:**
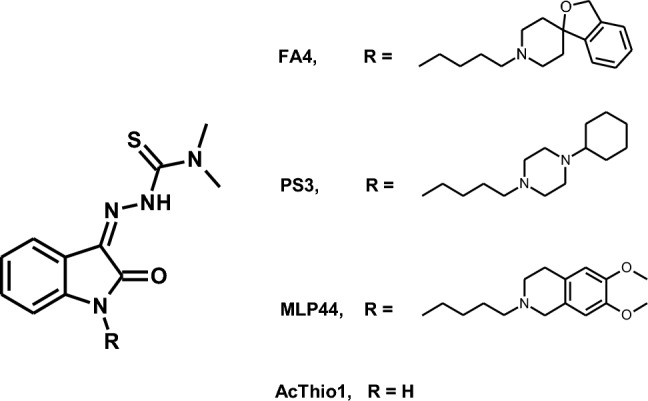
Structures of thiosemicarbazones (TSCs) investigated in the present study

Importantly, it has been shown that in the compound **ACThio1** {(Z)-*N,N*-dimethyl-2-(2-oxoindolin-3-ylidene)hydrazinecarbothioamide} (Fig. 1) [15] the sole *N,N*-dimethylthiosemicarbazone chelating moiety is sufficient to confer a cytotoxic action; on the other hand, the presence of the sigma-2 receptor-targeting moiety (as in the compounds **MLP44**, {(*Z*)-2-(1-(4-(6,7-dimetoxy-3,4-dihydroisoquinolin-2(1*H*)-yl)butyl)-2-oxoindolin-3-ylidene)-*N,N*-dimethylhydrazinecarbothioamide} and **PS3** {(*Z*)-2-[1-[4-(4-cyclohexylpiperazin-1-yl)butyl]-2-oxoindolin-3-ylidene]-*N,N*-dimethylhydrazinecarbothioamide}) resulted in the activation of different cell death pathways and more specific delivery to tumors, leading to reduced off-target effects [8] (Fig. 1). These results encouraged the production of a novel sigma-2 receptor-binding thiosemicarbazone named FA4 {(*Z*)-2-(1-(4-(3*H*-spiro[isobenzofuran-1,4′-piperidine]-1′-yl)butyl)-2-oxoindolin-3-ylidene)-*N,N*-dimethylhydrazinecarbothioamide} (Fig. 1), [9] whose structure was inspired by siramesine, a sigma-2 receptor reference compound which is cytotoxic in a number of cells, via pathways leading to oxidative stress induced by lysosomal leakage and mitochondrial destabilization [16–18]. This innovative compound performed better than the other TSCs in all pancreatic cancer cell lines tested, with particular cytotoxic activity against the aggressive gemcitabine-resistant human PANC-1 cell line [19]. In challenge with the other TSCs, the type/presence of diverse sigma-2 receptor-binding basic moieties were found to trigger different pathways in different cells [9] providing hints for application towards personalized therapies.

Based on the above, in this work, we studied the effect in MCF7 and A549 cells of all sigma-2 receptor-targeting TSCs (MLP44, PS3, and FA4, Fig. 1) and the non-targeted TSC (ACThio1, Fig. 1), with a focus on apoptotic pathways, such as caspase activation and the ER and mitochondrial stress. While all TSCs displayed an important cytotoxic effect in these cells and pancreatic cells, [9] FA4 was again the most promising TSC. These in vitro results suggest that FA4 is a promising MTDL for treating lung and breast cancer.

## Materials and methods

*Compounds*: The syntheses of FA4, MLP44, PS3, and ACThio1 were performed according to the procedures already reported [3, 9].

### Cell culture

Human MCF7 breast adenocarcinoma (#HTB-22) and human A549 carcinoma (#CCL-85) cell lines, non-tumor human breast (MCF10A, #CRL-10317) and lung (BEAS-2B, #CRL-9482) cells were obtained from American Type Culture Collection (ATCC, Bethesda, MD). MCF7 cells were cultured in DMEM medium (Gibco Thermo Scientific, Waltham, MA; #10,565,018), A549 in HAM’s F12 medium (Gibco Thermo Scientific; #11,765,054), and BEAS-2B in RPMI-1640 medium (Gibco Thermo Scientific; # 61,870,010), all supplemented with 10% v/v fetal bovine serum (FBS; Sigma, St. Louis, MO; #F4135) and 1% v/v penicillin–streptomycin (Sigma; #P4458). MCF10A cells were cultured in DMEM/F12 medium (Gibco Thermo Scientific; #31,331,028) supplemented with 5% v/v horse serum (Sigma; #H1270) and 1% v/v penicillin–streptomycin, 20 ng/mL epidermal growth factor (Sigma, #E4127), 10 µg/mL insulin (Sigma, #I3536), 0.5 mg/mL hydrocortisone (Sigma, #H0888), 100 ng/mL cholera toxin (Sigma, #C8052). Cells were kept in a humidified incubator at 37 °C with 5% CO_2_.

### Cell viability

Cell growth was determined using the MTT assay at 48 h [11, 20]. On day 1, 25,000 cells/well were seeded into 96-well plates in a volume of 100 μL. On day 2, the various drug concentrations (1 μM–100 μM) were added. In all experiments, the various drug solvents (EtOH, Sigma, # E7148; DMSO, Sigma, # D8418) were added to each control to evaluate possible cytotoxicity of the solvent. After the established incubation time with drugs (48 h), MTT (0.5 mg/mL, Sigma # M2003) was added to each well, and after 3–4 h incubation at 37 °C, the supernatant was removed. Formazan crystals were solubilized using 100 μl of DMSO/EtOH (1:1) and absorbance values at 570 and 630 nm were determined on the microplate reader Victor 3 from PerkinElmer Life Sciences (Waltham, MA).

### Caspase 3, 7 and 9 activity

Activation of caspase 3, caspase 7 and caspase 9 was measured using the Caspase 3/7 Fluorescence Assay kit (Cayman Chemical, Ann Arbor, MI; #10,009,135) and the Caspase 9 fluorimetric assay kit (Enzo Life Science, Roma, Italy; #ALX-850-224). The results were expressed as nmoles of the hydrolyzed substrate of each caspase/mg cellular proteins, according to a previously set titration curve.

### qRT-PCR

1 μg total RNA was reverse-transcribed using the iScript Reverse Transcription Supermix kit (Bio-Rad Laboratories, Hercules, CA; #1,708,840), according to the manufacturer’s instruction. 25 ng cDNA were amplified with 10 μL IQ^™^ SYBR Green Supermix (Bio-Rad Laboratories, # 1708880B05). Primers were designed with the qPrimer Depot software (http://primerdepot.nci.nih.gov/). qRT-PCR was carried out with a CFX96^™^ Real-Time Detector System (Bio-Rad Laboratories). Cycling conditions were: 30 s at 95 °C, followed by 40 cycles of denaturation (15 s at 95 °C), annealing/extension (30 s at 60 °C). The same cDNA preparation was used to quantify the genes of interest and the housekeeping gene *S14*, used to normalize gene expression. The relative quantitation of each sample was carried out using the Gene Expression Quantitation software (Bio-Rad Laboratories). Results were expressed in arbitrary units. For each gene, the expression in untreated cells was assumed to be “1”.

### Immunoblotting

Cells were rinsed with ice-cold lysis buffer (50 mM, Tris, 10 mM EDTA, 1% v/v Triton-X100), supplemented with the protease inhibitor cocktail set III (80 μM aprotinin, 5 mM bestatin, 1.5 mM leupeptin, 1 mM pepstatin; Sigma, # 535,140), 2 mM phenylmethylsulfonyl fluoride (Sigma: #P7626) and 1 mM Na_3_VO_4_, (Sigma; #S6508) then sonicated and centrifuged at 13,000 × g for 10 min at 4 °C. 20 μg protein extracts were subjected to SDS–PAGE and probed with the antibodies for: GRP78 (#ab21685), ATF6 (#ab122897), IRE-1 (#ab37073), PERK (#ab65142), actin (#ab1801) (all from Abcam, Cambridge, UK), followed by a peroxidase-conjugated secondary antibody (Bio-Rad Laboratories). The membranes were washed with Tris-buffered saline-Tween 0.1% v/v solution, and the proteins were detected by enhanced chemiluminescence (Bio-Rad Laboratories, #1,705,061). The densitometric analysis was performed using the ImageJ software (https://imagej.nih.gov/), calculating the ratio protein of interest/actin band density, putting actin band density as 1 relative unit in each condition. Densitometric analysis and whole blots are included as Supporting Information.

### Mitochondrial and total ROS measurement

Intramitochondrial reactive oxygen species (ROS) were measured using the MitoSOX fluorescent probe (Invitrogen ThermoScientific, # M36008), according to the manufacturer’s instructions. For total ROS, cells were incubated with 5 µmol/L of 5-(and-6)-chlorometyl-20,70-dichlorodihydro-fluorescein diacetate-acetoxymethyl ester (DCFDA-AM, Sigma, #21,884), as described by Riganti et al. 2015 [21]. The results are expressed as nmoles/mg mitochondrial or cellular proteins, respectively.

### Mitochondria depolarization

Staining with the JC-1 fluorescent probe (Biotium Inc., Freemont, CA; #30,001-30001-T) was performed as described in Riganti et al. 2015 [21]. Fluorescence units were used to calculate the percentage of green-fluorescent (i.e., depolarized) mitochondria vs red-fluorescent (i.e., polarized) mitochondria.

### Statistical analysis

Data were analyzed by applying the one-way measures analysis of variance (ANOVA) and Bonferroni’s multiple comparison tests as post hoc test. Results were reported as mean ± standard deviations (SD) of at least two or three independent experiments, performed in triplicate. Statistical significance was accepted at *p* < 0.05 and represented as: *p < 0.05, **p < 0.01, ***p < 0.001. The detailed statistical report is included as Supporting Information in Tables S2, S3, S4, S5 and S6.

## Results

### Density of sigma-2 receptors and binding affinity of TSCs in normal cells and in lung and breast cancer cells

While the affinity at sigma-2 receptors and the activity at P-gp of all TSCs is reported in Table S1 (Supplementary Information), the presence of the sigma-2 receptor was evaluated by flow cytometry in normal cells (BEAS-2B and MCF10A) and in lung (A549) and breast (MCF7) cancer cells. Cells were incubated with 100 nM of the selective sigma-2 fluorescent ligand NO1 [5, 22], specific binding was assessed by saturation of sigma-2 receptors; non-specific binding by DTG displacement in A549 and MCF7 cells. MCF10A and BEAS-2B were treated with 100 nM NO1, alone or with a saturating amount (10 µM) of DTG, determined in the respective tumoral counterpart. The results indicated that the sigma-2 receptor is ~ twofold more abundantly expressed in tumor (MCF7 and A549) than in normal cells (MCF10A and BEAS-2B). Moreover, sigma-2 receptor is more expressed in MCF7 than in A549 cancer cells (Fig. S1A, B, Supplementary Information). The detailed statistical report is provided in the supplementary information, Table S2.

### Cytotoxic activity of TSCs in normal cells and lung and breast cancer cells

The cytotoxic activity of the thiosemicarbazones FA4, MLP44, PS3, and ACThio1 was evaluated in human lung and breast normal and cancer cells (Table 1). The results showed that FA4 and PS3 showed low micromolar cytotoxic activities in the two tumor cell lines investigated (EC_50_ ranging from 1.53 to 1.84 μM for FA4 and from 1.81 to 2.20 μM for PS3). Moreover, in tumor MCF7 and A549 cell lines, the activity of FA4 and PS3 was 3–16-fold higher compared to the activity of MLP44 and ACThio1. The cytotoxicity of all TSCs was also evaluated in normal cells (MCF10A and BEAS-2B), where FA4 and PS3 showed a reduced activity concerning cancer cells (in line with the sigma-2 receptor density, Fig. S1A), whereas MLP44 and ACThio1 showed comparable activities. While off-target effects for TSCs cannot be excluded, for ACThio1, which lacks the sigma-2 receptor-targeting moiety, this behavior was quite expected and underscores the importance of the targeting moiety for the delivery of cytotoxic agents to cancer cells.

**Table 1:** Activity of TSCs in breast and lung cell lines

Cmpd	EC_50_ ± SEM^a^ (μM)			
	Tumor cells		Normal cells	
	MCF7	A549	MCF10A	BEAS-2B
FA4	1.53 ± 0.3	1.84 ± 0.4	4.31 ± 0.7	4.47 ± 1.1
MLP44	24.6^b^	10.4^b^	24.20 ± 0.8	11.9 ± 2.1
PS3	1.81^b^	2.20^b^	3.03 ± 0.6	6.22 ± 1.3
ACThio1	5.88 ± 1.1	4.08 ± 0.4	2.09 ± 0.4	3.39 ± 0.8

^a^Values represent the mean of *n* ≥ 3 separate experiments in duplicate ± SEM

^b^See Ref [3]

### Evaluation of the ability of TSCs to induce apoptosis in cancer cells

**Fig. 2:**
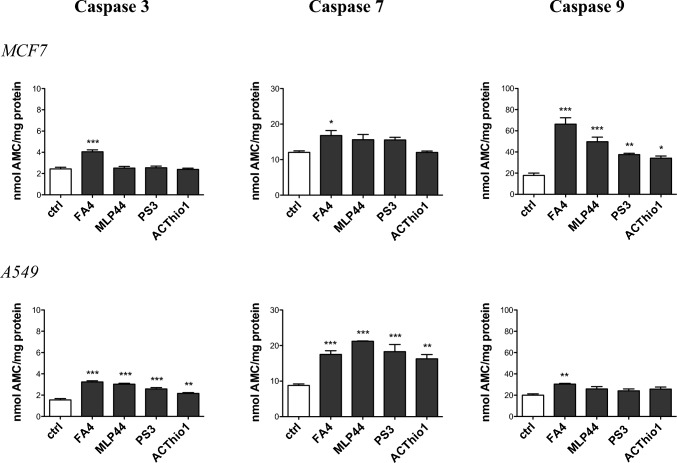
Activation of caspases 3, 7, and 9 by TSCs in breast and lung cancer cells. Fluorimetric measurement of caspases 3, 7, and 9 in cells treated for 2 h with 50 µM of each compound. Results are means ± SEM (*n* = 3). *p < 0.05, **p < 0.01, ***p < 0.001: vs ctrl cells (one-way ANOVA, Bonferroni test)

To investigate whether cytotoxicity induced by different TSCs leads to apoptotic cell death in lung and breast cancer cells, we first measured the activity of caspase 3, which irreversibly commits cells towards apoptosis, in MCF7 and A549 treated for 2 h with 50 μM solutions of the sigma-2 receptor-targeting TSCs FA4, MLP44, PS3 and the metal chelator ACThio1. In agreement with the previous finding [9], FA4 resulted to be the most potent inducer of caspase 3 in both cell lines compared to the other TSCs. The other compounds activated caspase 3 in A549 cells, but not in MCF7 cells (Fig. 2).

To better elucidate the apoptotic mechanism of TSCs, we analyzed the activity of two upstream caspases, namely, caspase 7, which is activated by the stress of endoplasmic reticulum (ER), where sigma-2 receptor is mainly localized [23], and caspase 9, which is typically activated following oxidative damage and depolarization of mitochondria, another site of sigma-2 receptor, elicited by TSCs [9]. Again, FA4 was the only TSC to activate both caspase 7 and caspase 9 in breast and lung cancer cell lines. By contrast, the other TSCs activated caspase 7 in MCF7 and A549 cells and caspase 9 in MCF7 cells (Fig. 2). The detailed statistical report is provided in the supplementary information, Table S3.

**Fig. 3:**
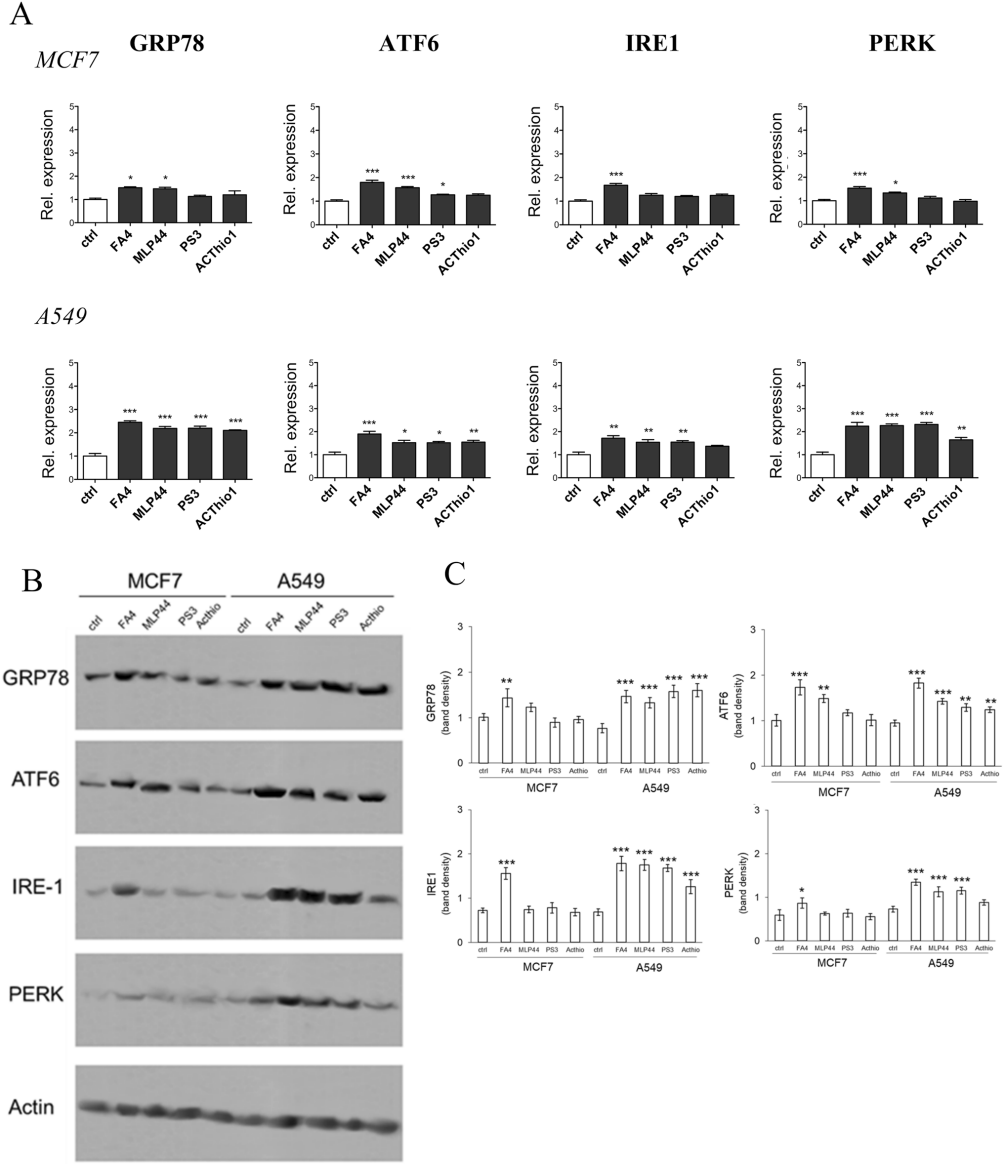
ER markers in TSC-treated breast and lung cancer cells. **A** mRNA expression of ER stress markers, measured by RT-PCR, in cells treated for 2 h with 50 µM solutions of each compound. Results are means ± SEM (*n* = 3). *p < 0.05, **p < 0.01, ***p < 0.001: vs ctrl cells (one-way ANOVA, Bonferroni test). **B** Representative immunoblot of the indicated proteins. Actin was used as control of equal protein loading. **C** Densitometric analysis of immunoblots. Data are presented as relative band density (mean of 3 independent experiments ± SD), corresponding to the ratio of the density of each band/density of corresponding actin band. *p < 0.05, **p < 0.01, ***p < 0.001: treated cells vs respective untreated (ctrl) cells (one-way ANOVA, Bonferroni test)

ER, stress causes the so-called unfolded protein response (UPR), which is perceived by the chaperone glucose-regulated protein 78 (GRP78) and by their downstream sensors: activating factor 6 (ATF6), inositol-requiring enzyme-1α (IRE-1α) and PKR-like ER kinase (PERK). If ER stress is brief and reversible, these sensors lead to cell survival; in case of persistent stress, the sensors trigger the activation of caspases 7/3 and subsequent apoptotic cell death [24]. The expression of ER stress markers was evaluated in MCF7 and A549 cancer cells (Fig. 3A–C; Fig. S2, Supplementary Information).

GRP78, ATF6, IRE1, and PERK increased upon FA4 treatment in both cell lines. Consistent with the prevalent activation of caspase 7, the other TSCs increased the expression of the four ER stress markers in A549 cells, while in MCF7 cells MLP44 and PS3 produced modest effects. The non-targeted compound ACThio1 had no or negligible effect, except A549 cells. The detailed statistical report is provided in the supplementary information, Tables S4 and S5.

**Fig. 4:**
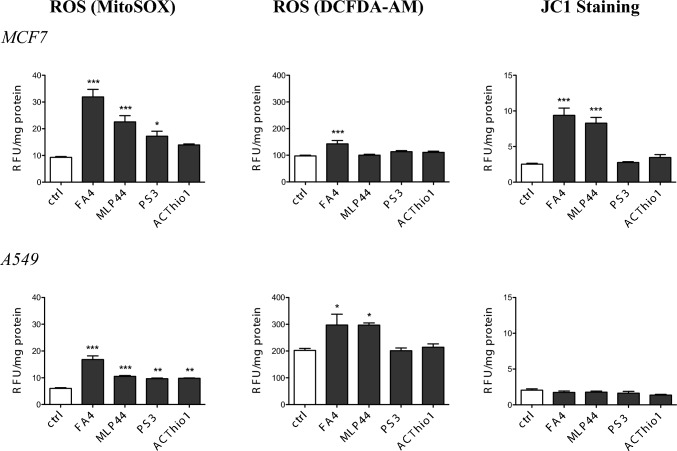
ROS and mitochondrial damage markers in TSC-treated breast and lung cancer cells. Fluorimetric staining of mitochondrial ROS (MitoSOX staining, **A**), whole cell ROS (DCFDA-AM probe, **B**), and mitochondrial depolarization (JC1 staining, **C**) in cells treated for 2 h with 50 µM of each compound. Results are means ± SEM (*n* = 3). *p < 0.05, **p < 0.01, ***p < 0.001: vs ctrl cells (one-way ANOVA, Bonferroni test)

Next, we focused on mitochondrial redox balance and polarization, as alteration of calcium homeostasis in mitochondria-associated ER membranes (MAM) increases ROS production and induces mitochondrial depolarization followed by cell death triggered by the caspase 9/caspase 3 axis [25]. Interestingly, FA4 and MLP44—and to a lesser extent PS3 and ACThio1—increased mitochondrial ROS both in MCF7 and A549 cancer cells (Fig. 4), with greater effects on the former, where they effectively induced mitochondrial damage-dependent caspase 9 (Fig. 2). In A549 cells, the increase in mitochondrial ROS, although present, was lower, probably as a consequence of the intrinsically high antioxidant defenses of these cells [26]. ROS levels in whole cells reflected those of mitochondrial ROS, being higher in FA4-treated cells, variable in MLP44-treated cells, and not significantly increased in PS3- and ACThio1-treated cells (Fig. 4). Notably, the higher the level of mitochondrial ROS, the greater the damage on the mitochondria, as assessed by mitochondrial depolarization with JC-1 staining (Fig. 4). Indeed, FA4 and MLP44 induced a strong depolarization in MCF7 cells, which are more susceptible to mitochondrial damage, compared to A549 cells, which are instead totally insensitive. Again, PS3 and ACThio1, which mildly increased mitochondrial ROS, did not produce any detectable changes in mitochondrial polarization. The detailed statistical report is provided in the supplementary information, Table S6.

These results explain caspase 9 activation in MCF7 cells that followed this rank order: FA4 > MLP44 > PS3 > ACThio1 (Fig. 2).

## Discussion

Multitarget TSCs that bind sigma receptors and coordinate metals have shown encouraging results in pancreatic cancer cell models both in vitro and in vivo [9]. Here, we extended the investigation of the biochemical mechanisms activated by TSCs in lung (A549) and breast (MCF7) cancer cells. In all cell lines, the presence of sigma-2 receptors was evaluated by flow cytometry. We found that the expression of sigma-2 receptors was ~ twofold higher in tumors (MCF7 and A549) than in normal cells (MCF10A and BEAS-2B). In particular, FA4 and PS3 exhibited low micromolar cytotoxic activities, 3–16-fold higher than the cytotoxicity of MLP44 and ACThio1 in all cell lines studied. Moreover, FA4 and PS3 showed reduced activity in normal (immortalized) breast and lung cells compared to the corresponding cancer cells, opening a putative therapeutic window for their potential use in vivo as cytotoxic agents more active against tumor cells than against untransformed cells. ACThio1, which is devoid of the sigma-2 receptor-targeting moiety (i.e., the basic amine), is instead characterized by an enhanced activity in normal cells compared to cancer cells, thus highlighting the importance of the basic moiety for reduced toxic action. The results from cytotoxicity assays prompted us to evaluate the possible apoptotic pathways induced by the four TSCs (FA4, MLP44, PS3, and ACThio1) in lung and breast cancer cell lines. Analysis of the ER-dependent (ER stress sensor, caspase 7/caspase 3 axis) and mitochondria-dependent (mitochondrial ROS and depolarization, caspase 9/caspase 3 axis) apoptotic pathways revealed variable activation by TSCs, which depended also on the cell line used. Lung cancer cells appeared more prone to apoptosis following ER stress, whereas breast cancer cells were more sensitive to mitochondrial oxidative stress and apoptosis.

The results also showed that all TSCs activated ER-dependent pro-apoptotic pathways in the lung cancer cell line, while FA4 and MLP44 also activated mitochondria-dependent pathways, in both lung and breast cancer cells. At least two hypotheses can be formulated to explain these differences. On one hand, differences in the structure of TSCs (i.e., different basic moieties) lead to the differential activation of ER- and/or mitochondria-dependent pathways, while the absence of such a moiety (i.e., ACThio1) results in higher toxicity and a reduced effect on caspase activation. On the other hand, the differences between the lung and breast cancer cell lines could be due to the different expression and localization of the sigma-2 receptors: while the potent sigma-2 receptor-targeting compound FA4 exerts similar effects, the other TSCs, characterized by lower cytotoxic potential, preferentially activate one apoptotic pathway, depending on the different expression/localization of sigma-2 receptors within each cell line.

## Conclusions

Overall, with the TSCs herein studied we have shown that while the presence of the sigma-2 receptor-targeting moiety results in more specific tumor delivery and activity, the peculiarities in the activated pathways that lead to cytotoxicity deserve to be investigated more in depth. The promising antitumor profile of FA4 in particular, makes it worthy of further studies, also from the perspective of specific therapies for each tumor type.

### Supplementary Information

Below is the link to the electronic supplementary material.

Supplementary file1 (DOCX 728 KB)

## Data Availability

All data generated during this study are included in this article and in the supplementary information file. Protocols for flow cytometry studies and for Calcein-AM assay and details for statistical analyses are reported in the supplementary information.

## Abbreviations

ACThio1: {(Z)-*N,N*-dimethyl-2-(2-oxoindolin-3-ylidene)hydrazinecarbothioamide}
A549: Lung cancer cells
ATF6: Activating factor 6
BEAS-2B: Normal lung cells
DCFDA-AM: 5-(and-6)-chlorometyl-20,70-dichlorodihydro-fluorescein diacetate-acetoxymethyl ester
DTG: 1,3-Di-ortho-tolylguanidine
ER: Endoplasmic reticulum
FA4: {(*Z*)-2-(1-(4-(3*H*-Spiro[isobenzofuran-1,4′-piperidine]-1${\prime}$-yl)butyl)-2-oxoindolin-3-ylidene)-*N,N*-dimethylhydrazinecarbothioamide}
FBS: Fetal bovine serum
FLU: Mean fluorescent units
GRP78: Chaperone glucose-regulated protein 78
IRE-1α: Inositol-requiring enzyme-1α
MAM: Mitochondria-associated ER membranes
MCF7: Breast cancer cells
MCF10A: Normal breast cells
MLP44: {(*Z*)-2-(1-(4-(6,7-Dimethoxy-3,4-dihydroisoquinolin-2(1*H*)-yl)butyl)-2-oxoindolin-3-ylidene)-*N,N*-dimethylhydrazinecarbothioamide}
MTDL: Multi target directed ligands
PERK: PKR-like ER kinase
P-gp: P-glycoprotein
PS3: {(*Z*)-2-[1-[4-(4-Cyclohexylpiperazin-1-yl)butyl]-2-oxoindolin-3-ylidene]-*N,N*-dimethylhydrazinecarbothioamide}
ROS: Reactive oxygen species
SD: Standard deviation
SEM: Standard error of the mean
TSCs: Multifunctional thiosemicarbazones
UPR: Unfolded protein response
