# Supplementary material for: Cytotoxic pathways activated by multifunctional thiosemicarbazones targeting sigma-2 receptors in breast and lung carcinoma cells
Source: Pharmacol Rep. 2023 Oct 5;75(6):1588–96. doi: 10.1007/s43440-023-00531-y (PMC10661773; doi:10.1007/s43440-023-00531-y)
Supplement: Supplementary file 1 — Supplementary file1 (DOCX 728 KB) [file 43440_2023_531_MOESM1_ESM.docx]

**Supplementary Information**

**Cytotoxic pathways activated by multifunctional thiosemicarbazones targeting sigma-2 receptors in breast and lung carcinoma cells**

Joanna Kopecka^a^, Alessandra Barbanente^b^, Daniele Vitone^b^, Fabio Arnesano^b^, Nicola Margiotta^b^, Paola Berchialla^c^, Mauro Niso^d^, Chiara Riganti^a*^, Carmen Abate^d,e*^

^a^Department of Oncology, University of Turin, via Nizza 44, 10126, Torino, Italy.

^b^Università degli Studi di Bari Aldo Moro, Dipartimento di Chimica, Via Orabona 4, 70125 Bari.

^c^ Department of Clinical and Biological Sciences, University of Turin, via Santena5/bis, 10126 Torino.

^d^Università degli Studi di Bari Aldo Moro, Dipartimento di Farmacia-Scienze del Farmaco, Via Orabona 4, 70125 Bari.

^e^Consiglio Nazionale delle Ricerche (CNR), Istituto di Cristallografia, Via Amendola, 70125 Bari, Italy.

^*^ corresponding authors.

**Content**: Protocol for flow cytometry studies; Fig, S1: Density of sigma-2 receptors in breast and lung cells, evaluated by flow cytometry; Protocol for the evaluation of the activity of drugs at P-gp through the Calcein-AM assay; Table S1, Values of binding affinity of TSCs and reference compounds towards sigma-2 receptors and P-gp activity; Fig, S2: Whole blot of Figure 3B; Table S2-S6: Statistical report for all data.

**Flow cytometry studies**

Flow cytometry studies to detect sigma-2 receptors density was carried out according to Niso et al, [9]. In order to detect the sigma-2 receptor content, MCF7 and A549 cells were incubated with or without 10 µM of reference compound DTG, followed by 100 nmol/L of sigma-2 fluorescent compound (NO1, 2-{6-[2-(3-(6,7-dimethoxy-3,4-dihydroisoquinolin-2(1*H*)-yl)propyl)-3,4-dihydroisoquinolin-1(2*H*)-one-5-yloxy]hexyl}-5-(dimethylamino)isoindoline-1,3-dione) [5,22] for 75 min at 37 °C. To mask sigma-1 receptors, (+)-pentazocine (10 µM) was co-incubated. At the end of the incubation periods, the cells were washed twice with PBS, detached with 200 µl Cell Dissociation Solution (Sigma Chemical Co,) for 10 min at 37 °C, centrifuged at 13,000 g for 5 min and resuspended in 500 µl PBS. Fluorescence was recorded using a Bio-Guava® easyCyte™ 5 Flow Cytometry System (Millipore, Billerica, MA, USA) equipped with a 530 nm band pass filter. For each analysis, 50,000 events were collected and analyzed using InCyte software (Millipore).

**Fig. S1** *Density of sigma-2 receptors in breast and lung cells, evaluated by flow cytometry (n=3)*. (A) Mean fluorescence units (FLU) of cells incubated with 100 nM **NO1,** ***p<0,001: tumor vs normal cells (one way ANOVA, Bonferroni test). (B) Representative histograms of sigma-2 receptors measured in cells grown in the absence (ctrl) or presence of 100 nM **NO1**, alone or with different concentrations of DTG.

A

B


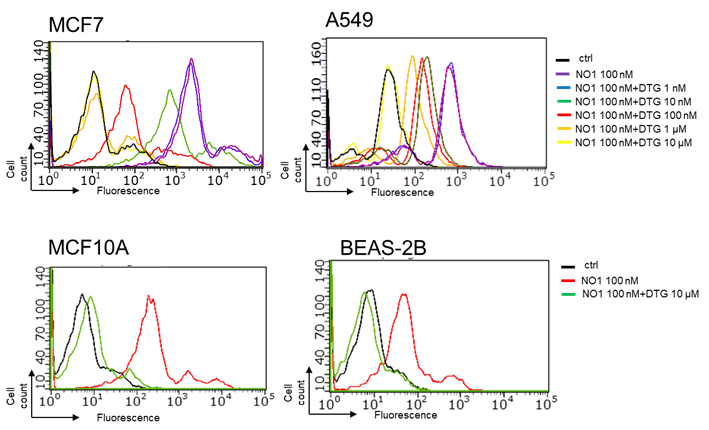


**Calcein-AM assay**

According to a previously reported procedure [8], MDCK-MDR1 cell lines (50,000 cells per well) were seeded into black CulturePlate 96/wells plate with 100 mL medium and grown overnight to confluence. Aliquots (100 μL) of tested compounds in culture medium, at scalar concentrations ranging from 0,1 to 100 μM, were added to each well. The plate was incubated at 37 °C for 30 min, Calcein-AM in phosphate buffered saline (PBS, 100 mL) was added to each well at a final concentration of 2,5 mM, and the plate was incubated for 30 min. The plate was washed 3 times with 100 mL ice cold PBS, Saline buffer (100 mL) was added to each well and the plate was read by a PerkinElmer Victor3 spectrofluorimeter at excitation and emission wavelengths of 485 nm and 535 nm, respectively. Under these conditions, calcein cell accumulation in the absence and in the presence of tested compounds was evaluated, and a fluorescence basal level was estimated by untreated cells. In treated wells, the increase of fluorescence with respect to the basal level was measured, EC_50_ values were determined by fitting the fluorescence increase percentage versus log[dose] with GraphPad Prism Software 5,0 (GraphPad Software, Inc,: San Diego, CA).

**Table S1,** *Values of binding affinity of TSCs and reference compounds towards sigma-2 receptors and P-gp activity.*

| **Cmpd** | **Sigma-2 Affinity (nM)** | **P-gp Activity (μM)** |
| --- | --- | --- |
|  | *K*_i_ ± SEM^a^ | EC_50_ ± SEM^a^ |
|  |  |  |
| **FA4** | 15,8^b^ | 3,8±0,6 |
| **MLP44** | 34,1^c^ | 3,04 ^c^ |
| **PS3** | 35,4^c^ | 2,83 ^c^ |
| **AcThio1** | >10000^d^ | Not Active^d^ |
| **DTG** | 22,5±1,2 |  |

^a^Values represent the mean of n ≥ 3 separate experiments in duplicate ± SEM (where applicable); ^b^[9]; ^c^[3]; ^d^[8].

**Fig, S2.** *Whole blot of Figure 3B.*


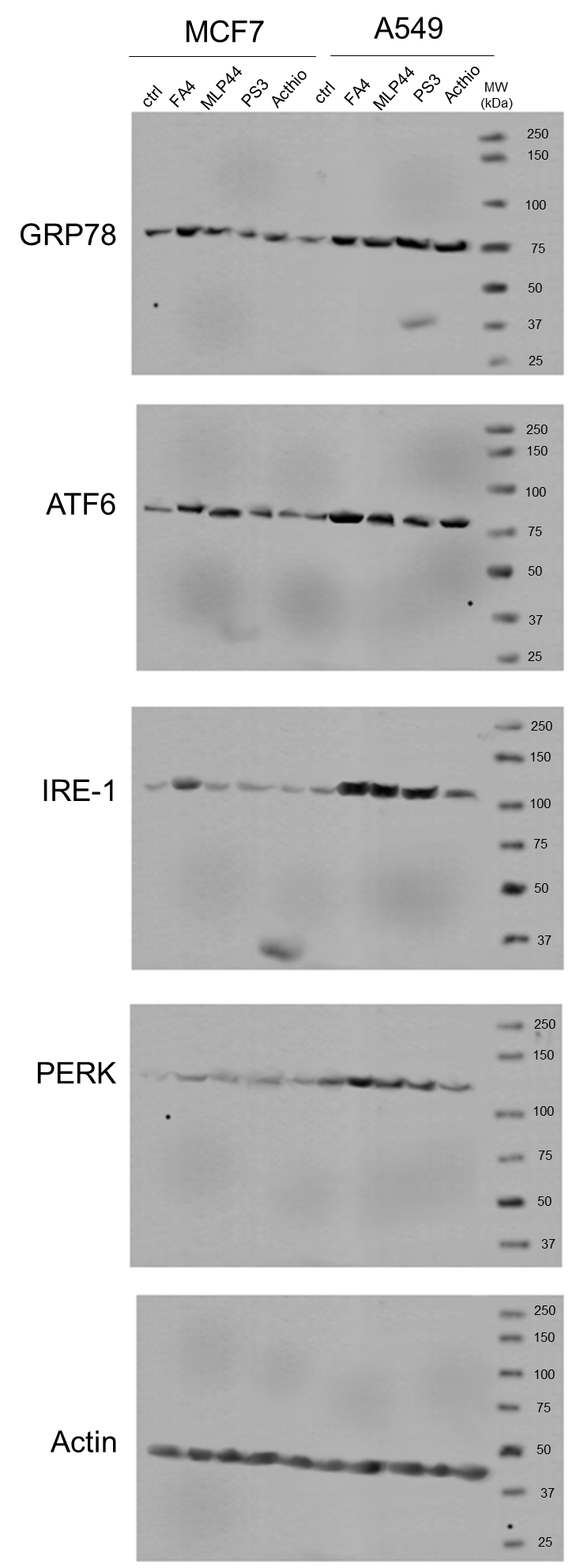


**Table S2. Statistical summary of data reported in Supplementary Figure S1A.** Results of one-way ANOVA for data presented in Figure S1A.

| One-way analysis of variance |  |  |  |
| --- | --- | --- | --- |
| P value | < 0,0001 |  |  |
|  | *** |  |  |
|  | Yes |  |  |
| P value summary | 4 |  |  |
| Are means signif, different? (P < 0,05) | 1696 |  |  |
| R square | 0,9992 |  |  |
| Bonferroni's Multiple Comparison Test |  |  |  |
| ANOVA Table | SS | df | MS |
| Treatment (between columns) | 35350 | 3 | 11783 |
| Residual (within columns) | 27,79 | 4 | 6,946 |
| Total | 35378 | 7 |  |

**Table S3. Statistical summary of data reported in Figure 2.** Results of one-way ANOVA for data presented in Figure 2.

| **MCF7** | **Caspase 3** |  |  | **Caspase 7** |  |  | **Caspase 9** |  |  |
| --- | --- | --- | --- | --- | --- | --- | --- | --- | --- |
| One-way analysis of variance |  |  |  |  |  |  |  |  |  |
| P value | < 0,0001 |  |  | 0,0093 |  |  | < 0,0001 |  |  |
| P value summary | *** |  |  | ** |  |  | *** |  |  |
| Are means signif, different? (P < 0,05) | Yes |  |  | Yes |  |  | Yes |  |  |
| Number of groups | 5 |  |  | 5 |  |  | 5 |  |  |
| F | 22,89 |  |  | 4,983 |  |  | 24,68 |  |  |
| R square | 0,8592 |  |  | 0,5706 |  |  | 0,8681 |  |  |
| Bonferroni's Multiple Comparison Test |  |  |  |  |  |  |  |  |  |
| ANOVA Table | SS | df | MS | SS | df | MS | SS | df | MS |
| Treatment (between columns) | 8,026 | 4 | 2,007 | 79,08 | 4 | 19,77 | 5241 | 4 | 1310 |
| Residual (within columns) | 1,315 | 15 | 0,08767 | 59,51 | 15 | 3,967 | 796,2 | 15 | 53,08 |
| Total | 9,341 | 19 |  | 138,6 | 19 |  | 6037 | 19 |  |
|  |  |  |  |  |  |  |  |  |  |
| **A549** |  |  |  |  |  |  |  |  |  |
| One-way analysis of variance |  |  |  |  |  |  |  |  |  |
| P value | < 0,0001 |  |  | < 0,0001 |  |  | 0,008 |  |  |
| P value summary | *** |  |  | *** |  |  | ** |  |  |
| Are means signif, different? (P < 0,05) | Yes |  |  | Yes |  |  | Yes |  |  |
| Number of groups | 5 |  |  | 5 |  |  | 5 |  |  |
| F | 39 |  |  | 16,01 |  |  | 5,181 |  |  |
| R square | 0,9123 |  |  | 0,8102 |  |  | 0,5801 |  |  |
| Bonferroni's Multiple Comparison Test |  |  |  |  |  |  |  |  |  |
| ANOVA Table | SS | df | MS | SS | df | MS | SS | df | MS |
| Treatment (between columns) | 7,394 | 4 | 1,849 | 343,7 | 4 | 85,92 | 224,3 | 4 | 56,08 |
| Residual (within columns) | 0,711 | 15 | 0,0474 | 80,51 | 15 | 5,367 | 162,3 | 15 | 10,82 |
| Total | 8,105 | 19 |  | 424,2 | 19 |  | 386,7 | 19 |  |

**Table S4. Statistical summary of data reported in Figure 3A.** Results of one-way ANOVA for data presented in Figure 3A.

| **MCF7** | **GRP78** |  |  | **ATF6** |  |  | **IRE1** |  |  | **PERK** |  |  |
| --- | --- | --- | --- | --- | --- | --- | --- | --- | --- | --- | --- | --- |
| One-way analysis of variance |  |  |  |  |  |  |  |  |  |  |  |  |
| P value | 0,0112 |  |  | < 0,0001 |  |  | 0,0002 |  |  | 0,0002 |  |  |
| P value summary | * |  |  | *** |  |  | *** |  |  | *** |  |  |
| Are means signif, different? (P < 0,05) | Yes |  |  | Yes |  |  | Yes |  |  | Yes |  |  |
| Number of groups | 5 |  |  | 5 |  |  | 5 |  |  | 5 |  |  |
| F | 5,784 |  |  | 27,72 |  |  | 16,15 |  |  | 15,86 |  |  |
| R square | 0,6982 |  |  | 0,9173 |  |  | 0,866 |  |  | 0,8638 |  |  |
| Bonferroni's Multiple Comparison Test |  |  |  |  |  |  |  |  |  |  |  |  |
| ANOVA Table | SS | df | MS | SS | df | MS | SS | df | MS | SS | df | MS |
| Treatment (between columns) | 0,5631 | 4 | 0,1408 | 1,158 | 4 | 0,2894 | 0,7261 | 4 | 0,1815 | 0,6884 | 4 | 0,1721 |
| Residual (within columns) | 0,2434 | 10 | 0,02434 | 0,1044 | 10 | 0,01044 | 0,1124 | 10 | 0,01124 | 0,1085 | 10 | 0,01085 |
| Total | 0,8065 | 14 |  | 1,262 | 14 |  | 0,8385 | 14 |  | 0,797 | 14 |  |
|  |  |  |  |  |  |  |  |  |  |  |  |  |
| **A549** |  |  |  |  |  |  |  |  |  |  |  |  |
| One-way analysis of variance |  |  |  |  |  |  |  |  |  |  |  |  |
| P value | < 0,0001 |  |  | 0,001 |  |  | 0,0026 |  |  | < 0,0001 |  |  |
| P value summary | *** |  |  | *** |  |  | ** |  |  | *** |  |  |
| Are means signif, different? (P < 0,05) | Yes |  |  | Yes |  |  | Yes |  |  | Yes |  |  |
| Number of groups | 5 |  |  | 5 |  |  | 5 |  |  | 5 |  |  |
| F | 48,27 |  |  | 11,41 |  |  | 8,824 |  |  | 25,33 |  |  |
| R square | 0,9508 |  |  | 0,8202 |  |  | 0,7792 |  |  | 0,9102 |  |  |
| Bonferroni's Multiple Comparison Test |  |  |  |  |  |  |  |  |  |  |  |  |
| ANOVA Table | SS | df | MS | SS | df | MS | SS | df | MS | SS | df | MS |
| Treatment (between columns) | 3,854 | 4 | 0,9634 | 1,232 | 4 | 0,308 | 0,8994 | 4 | 0,2248 | 3,891 | 4 | 0,9729 |
| Residual (within columns) | 0,1996 | 10 | 0,01996 | 0,27 | 10 | 0,027 | 0,2548 | 10 | 0,02548 | 0,3841 | 10 | 0,03841 |
| Total | 4,053 | 14 |  | 1,502 | 14 |  | 1,154 | 14 |  | 4,276 | 14 |  |

**Table S5. Statistical summary of data reported in Figure 3C.** Results of one-way ANOVA for data presented in Figure 3C.

| **MCF7** | **GRP78** |  |  | **ATF6** |  |  | **IRE1** |  |  | **PERK** |  |  |
| --- | --- | --- | --- | --- | --- | --- | --- | --- | --- | --- | --- | --- |
| One-way analysis of variance |  |  |  |  |  |  |  |  |  |  |  |  |
| P value | 0,0012 |  |  | <0,0001 |  |  | <0,0001 |  |  | 0,0168 |  |  |
| P value summary | ** |  |  | **** |  |  | **** |  |  | * |  |  |
| Are means signif, different? (P < 0,05) | Yes |  |  | Yes |  |  | Yes |  |  | Yes |  |  |
| Number of groups | 5 |  |  | 5 |  |  | 5 |  |  | 5 |  |  |
| F | 10,66 |  |  | 21,87 |  |  | 42,91 |  |  | 5,102 |  |  |
| R square | 0,81 |  |  | 0,8974 |  |  | 0,9449 |  |  | 0,6711 |  |  |
| Bonferroni's Multiple Comparison Test |  |  |  |  |  |  |  |  |  |  |  |  |
| ANOVA Table | SS | df | MS | SS | df | MS | SS | df | MS | SS | df | MS |
| Treatment (between columns) | 0,6 | 4 | 0,15 | 1,22 | 4 | 0,3051 | 1,656 | 4 | 0,4139 | 0,1786 | 4 | 0,0447 |
| Residual (within columns) | 0,1407 | 10 | 0,0141 | 0,1395 | 10 | 0,014 | 0,09647 | 10 | 0,0096 | 0,08753 | 10 | 0,0088 |
| Total | 0,7408 | 14 |  | 1,36 | 14 |  | 1,752 | 14 |  | 0,2662 | 14 |  |
|  |  |  |  |  |  |  |  |  |  |  |  |  |
| **A549** | **GRP78** |  |  | **ATF6** |  |  | **IRE1** |  |  | **PERK** |  |  |
| One-way analysis of variance |  |  |  |  |  |  |  |  |  |  |  |  |
| P value | <0,0001 |  |  | <0,0001 |  |  | <0,0001 |  |  | <0,0001 |  |  |
| P value summary | **** |  |  | **** |  |  | **** |  |  | **** |  |  |
| Are means signif, different? (P < 0,05) | Yes |  |  | Yes |  |  | Yes |  |  | Yes |  |  |
| Number of groups | 5 |  |  | 5 |  |  | 5 |  |  | 5 |  |  |
| F | 21,64 |  |  | 48,93 |  |  | 41,5 |  |  | 27,45 |  |  |
| R square | 0,8964 |  |  | 0,9514 |  |  | 0,9432 |  |  | 0,9165 |  |  |
| Bonferroni's Multiple Comparison Test |  |  |  |  |  |  |  |  |  |  |  |  |
| ANOVA Table | SS | df | MS | SS | df | MS | SS | df | MS | SS | df | MS |
| Treatment (between columns) | 1,166 | 3 | 0,3888 | 1,234 | 4 | 0,3086 | 2,63 | 4 | 0,6576 | 0,7005 | 4 | 0,1751 |
| Residual (within columns) | 0,1204 | 8 | 0,0151 | 0,06307 | 10 | 0,0063 | 0,1585 | 10 | 0,0159 | 0,0638 | 10 | 0,0064 |
| Total | 1,287 | 11 |  | 1,297 | 14 |  | 2,789 | 14 |  | 0,7643 | 14 |  |

**Table S6. Statistical summary of data reported in Figure 4.** Results of one-way ANOVA for data presented in Figure 4.

| **MCF7** | **ROS (MitoSOX)** |  |  | **ROS**  **(DCFDA-AM)** |  |  | **JC1 Staining** |  |  |
| --- | --- | --- | --- | --- | --- | --- | --- | --- | --- |
|  |  |  |  |  |  |  |  |  |  |
| One-way analysis of variance |  |  |  |  |  |  |  |  |  |
| P value | < 0,0001 |  |  | 0,0015 |  |  | < 0,0001 |  |  |
| P value summary | *** |  |  | ** |  |  | *** |  |  |
| Are means signif, different? (P < 0,05) | Yes |  |  | Yes |  |  | Yes |  |  |
| Number of groups | 5 |  |  | 5 |  |  | 5 |  |  |
| F | 21,83 |  |  | 7,63 |  |  | 27,99 |  |  |
| R square | 0,8534 |  |  | 0,6705 |  |  | 0,8818 |  |  |
| Bonferroni's Multiple Comparison Test |  |  |  |  |  |  |  |  |  |
| ANOVA Table | SS | df | MS | SS | df | MS | SS | df | MS |
| Treatment (between columns) | 1212 | 4 | 303 | 5078 | 4 | 1269 | 172 | 4 | 42,99 |
| Residual (within columns) | 208,2 | 15 | 13,88 | 2496 | 15 | 166,4 | 23,04 | 15 | 1,536 |
| Total | 1420 | 19 |  | 7574 | 19 |  | 195 | 19 |  |
|  |  |  |  |  |  |  |  |  |  |
| **A549** |  |  |  |  |  |  |  |  |  |
| One-way analysis of variance |  |  |  |  |  |  |  |  |  |
| P value | < 0,0001 |  |  | 0,0037 |  |  | 0,172 |  |  |
| P value summary | *** |  |  | ** |  |  | ns |  |  |
| Are means signif, different? (P < 0,05) | Yes |  |  | Yes |  |  | No |  |  |
| Number of groups | 5 |  |  | 5 |  |  | 5 |  |  |
| F | 35,6 |  |  | 6,228 |  |  | 1,849 |  |  |
| R square | 0,9047 |  |  | 0,6242 |  |  | 0,3303 |  |  |
| Bonferroni's Multiple Comparison Test |  |  |  |  |  |  |  |  |  |
| ANOVA Table | SS | df | MS | SS | df | MS | SS | df | MS |
| Treatment (between columns) | 243,5 | 4 | 60,88 | 40131 | 4 | 10033 | 1,027 | 4 | 0,2568 |
| Residual (within columns) | 25,65 | 15 | 1,71 | 24163 | 15 | 1611 | 2,083 | 15 | 0,1388 |
| Total | 269,2 | 19 |  | 64294 | 19 |  | 3,11 | 19 |  |
